# Supplementary material for: The Role of Contextual Variables and Structural Diversity on College Students’ Engineering Self-Efficacy
Source: Behav Sci (Basel). 2024 Jul 4;14(7):564. doi: 10.3390/bs14070564 (PMC11274101; doi:10.3390/bs14070564)
Supplement: Supplementary file 1 [file behavsci-14-00564-s001.zip › behavsci-3062410-supplementary.pdf]

Demographic Information

Directions: *The following are some questions about you and your family. Please* ***fill in, check, OR circle*** *the best description of you and your family members.*

Your Age: (insert number)

Your Sex:

a. Female
b. Male
c. Trans Male/Trans Man
d. Trans Female/Trans Woman
e. Genderqueer/Gender Non-Conforming
f. Others (please specify): _________

Your ethnicity:

a. Asian or Asian American
b. Black or African American
c. Hispanic or Latinx
d. White, Caucasian, European, not Hispanic
e. American Indian
f. Multiracial/Multiethnic: parents are from two different groups
g. Other (please specify): _________

Class Standing:

a. Freshman
b. Sophomore
c. Junior
d. Senior
e. Other (please specify): _________

Please identify your major:

a. Chemical Engineering
b. Civil Engineering
c. Electrical and Computer Engineering
d. Engineering Physics
e. Engineering Technology
f. Industrial Engineering
g. Information and Communication Technology
h. Mechanical Engineering
i. Aerospace Engineering
j. Surveying Engineering
k. Other (please specify): _______

Engineering Self-Efficacy

Engineering Self-Efficacy Scale (ESE)
Lent, Brown, et al. (2005)

Instructions: The following is a list of major steps along the way to competing an engineering degree. Please indicate how much confidence you have in your ability to complete each of these steps in relation to the engineering major that you are most likely to pursue. Use the 0-9 scale below to indicate your degree of confidence. How much confidence do you have in your ability to:

	**No Confidence at all**			**Some Confidence**						**Much Confidence**
**1. Complete all of the "basic science" (i.e. math, physics, chemistry) requirements for your engineering major with grades of B or better.**	0	1	2	3	4	5	6	7	8	9
**2. Excel in your engineering major over the next semester**	0	1	2	3	4	5	6	7	8	9
**3. Excel in your engineering major over the next two semesters. Complete the upper level required courses in your engineering major**	0	1	2	3	4	5	6	7	8	9
**4. Complete the upper level required courses in your engineering major with an overall grade point average of B or better.**	0	1	2	3	4	5	6	7	8	9

Perceived Supports and Barriers in Engineering

Engineering Perceived Supports and Barriers Scale (ESBS)
Lent, Brown, et al. (2005)

Instructions: If you were to major in an engineering field, how likely would you be to...

	Not at all likely	A little likely	Moderately likely	Quite likely	Extremely likely
**1. Have access to a "role model" in the field (i.e., someone you can look up to and learn from observing).**	1	2	3	4	5
**2. Feel support for this decision from important people in your life (e.g., teachers).**	1	2	3	4	5
**3. Feel that there are people "like you" in this field.**	1	2	3	4	5
**4. Get helpful assistance from a tutor, if you felt you needed such help.**	1	2	3	4	5
**5. Get encouragement from your friends for pursing this major.**	1	2	3	4	5
**6. Get helpful assistance from your advisor.**	1	2	3	4	5
**7. Feel that your family members support this decision.**	1	2	3	4	5
**8. Feel that close friends or relatives would be proud of you for making this decision.**	1	2	3	4	5
**9. Have access to a "mentor" who could offer you advice and encouragement.**	1	2	3	4	5
**10. Receive negative comments or discouragement about your major from family members.**	1	2	3	4	5
**11. Worry that such as a career path would require too much time or schooling.**	1	2	3	4	5
**12. Feel that you don't fit in socially with other students in this major.**	1	2	3	4	5
**13. Receive negative comments or discouragement about your major from your friends.**	1	2	3	4	5
**14. Feel pressure from parents or other important people to change your major to some other field.**	1	2	3	4	5
